# Shaping healthier futures: community-level impact of the Alberta Healthy Communities Approach

**DOI:** 10.24095/hpcdp.46.3.02

**Published:** 2026-03

**Authors:** Christina Gillies, Lisa K. Allen-Scott, Courtney Baay, Nicole Frenette, Jacky Ka Kei Liu, Stephanie Patterson

**Affiliations:** 1 Public Health Evidence & Innovation, Primary Care Alberta, Calgary, Alberta, Canada; 2 School of Public Health, University of Alberta, Edmonton, Alberta, Canada; 3 Department of Community Health Sciences, University of Calgary, Calgary, Alberta, Canada; 4 Department of Oncology, University of Calgary, Calgary, Alberta, Canada; 5 Faculty of Social Work, University of Calgary, Calgary, Alberta, Canada

**Keywords:** built environment, chronic disease, primary prevention, evaluation, Healthy Communities Approach

## Abstract

**Introduction:**

Built environment initiatives that change the physical places in which people live, work and play offer an approach to preventing cancer and chronic diseases. The purpose of this study was to evaluate the effectiveness of the Alberta Healthy Communities Approach Phase II (AHCA II), a community-based approach to creating healthy environments within and across rural communities and addressing modifiable health behaviours to prevent and reduce cancer and chronic disease.

**Methods:**

Nineteen rural communities participated in AHCA II. Data collected with and by community members included two pre- and postimplementation assessment tools and postimplementation focus groups and surveys. Qualitative and quantitative data sources were triangulated to determine community-level outcomes and impacts.

**Results:**

The evaluation found three key outcomes and impacts of the AHCA: supportive (built) environments for health; community wellness culture; and community capacity. These intersecting categories demonstrate the positive effects of healthy community initiatives on improving the built environment and supporting health behaviours such as healthy eating, physical activity, ultraviolet radiation protection and tobacco reduction.

**Conclusion:**

In addition to improving supportive environments for health, the AHCA facilitated cultural changes and improved community capacity within and across rural communities in Alberta. Each of these components is required to support long-term behaviour changes that promote health and prevent cancer and chronic disease. While these results are encouraging, time and additional evaluations are required to determine whether behavioural changes are sustained and result in reduced rates of cancer and chronic disease.

**Highlights**

- The Alberta Healthy Communities Approach (AHCA) addresses multiple environmental factors and modifiable health behaviours to reduce cancer and chronic disease risks in rural communities.
- Multisectoral teams improved built environments for health through physical changes to community environments and programming to support healthy eating, physical activity, sun protection and tobacco reduction.
- The AHCA created a novel health and wellness culture within communities, which increased community engagement and participation in health-promoting initiatives.
- Communities increased their capacity to collectively promote health by identifying the determinants of health, improving multisectoral partnerships and leveraging community resources.

## Introduction

In Canada, the high prevalence of cancer, cardiovascular disease, diabetes and other chronic diseases^1^ challenge policy-makers, public health practitioners and communities to improve supportive environments for health. While interconnected environmental features and mediating factors influence individual health behaviours, rates of cancer and other chronic diseases may be improved by changing the physical environment to support healthy behaviours. For instance, clean, safe, walkable neighbourhoods with access to green spaces and affordable food outlets can encourage physical activity, healthy food choices and a sense of community belonging.^2^,^3^

As features of the built environment are not available equally within and across all communities, the built environment (broadly defined as the places and spaces in which people live, work and play on a daily basis^2^,^4^) can foster or exacerbate inequities in health outcomes.^3^,^4^ Although patterns differ based on health indicators, people living in rural areas experience health disadvantages and generally have worse health outcomes than their urban counterparts.^5^-^8^ For example, risk for obesity, cardiovascular disease and lung cancer are higher in rural areas.^8^-^10^ Marked variations across the urban–rural continuum influence prevalence of health risk factors, including demographic characteristics (e.g. age, income, education), health behaviours (e.g. smoking, healthy eating) and social and structural determinants of health (e.g. income, air pollution, and distribution of resources).^5^-^7^,^11^-^13^ At the same time, rural communities benefit from having more opportunities to form close social relationships, increased availability of social support and greater senses of belonging.^8^,^11^ Efforts to improve the built environment that recognize the unique characteristics and diversity of rural communities can help to improve rural health and reduce inequities in cancer and chronic disease. 

The Healthy Communities Approach offers a community- and settings-based approach to building and promoting supportive environments for health as well as cancer and chronic disease prevention.^14^ The movement addresses multiple determinants of health, including the built environment, through community engagement and multisectoral partnerships. In Canada, the Healthy Communities Approach has been implemented in smaller communities and large cities and towns alike through community-focused health initiatives.^14^,^15^ The movement prioritizes developing a community’s existing capacity to improve health and well-being and facilitating collaborative action to address issues of local relevance and significance.^15^

The Healthy Communities Approach in Canada has been adopted by partners in the public, non-profit and private sectors in several provinces and has persisted for more than 30 years.^15^ However, there are gaps in our knowledge concerning the healthy community initiatives that have been developed. There have also been few evaluations of the effectiveness of the approach in improving the built environment, health behaviours and health outcomes, or else evaluation findings are not freely accessible.^16^ In addition, less is known about initiatives to improve the built environment in rural communities than in urban contexts. Barriers to evaluation and knowledge-sharing exist both within and outside communities (e.g. lack of human and financial resources). However, determining the effectiveness of community-focused health initiatives in different contexts is important for informing preventive health programs and policies and for disseminating this information. 

The Healthy Communities Approach has been adapted for rural communities in Alberta and established as the evidence-based Alberta Healthy Communities Approach (AHCA).^17^ Intended for addressing multiple determinants of health, the AHCA has the potential to improve social, economic and environmental factors within and across communities, as well as modifiable health behaviours, in order to reduce cancer and chronic disease rates in the province. The purpose of this study was to evaluate the community-level outcomes and impacts of the AHCA.

## Methods

***Intervention***

The AHCA was co-designed and developed by the Communities Team in Cancer Prevention and Screening Innovation (CPSI), Primary Care Alberta (formerly Alberta Health Services) and collaborating partners who adapted the Healthy Communities Approach to the context of rural communities in Alberta in Phase I (2015–2019) and Phase II (2019–2023). Adaptations were based on the capacity and assets in rural contexts and were designed to support initiatives that address the determinants of health associated with cancer and chronic disease prevention, for example, built environments that promote physical activity and ultraviolet radiation (UVR) protection. 

The process of adapting and piloting the AHCA in Phase I has been described elsewhere.^17^ In this article, we report on AHCA Phase II (AHCA II), the aim of which was to determine the effectiveness and efficiency of the AHCA to inform sustainability and scaling.

**Figure 1:**
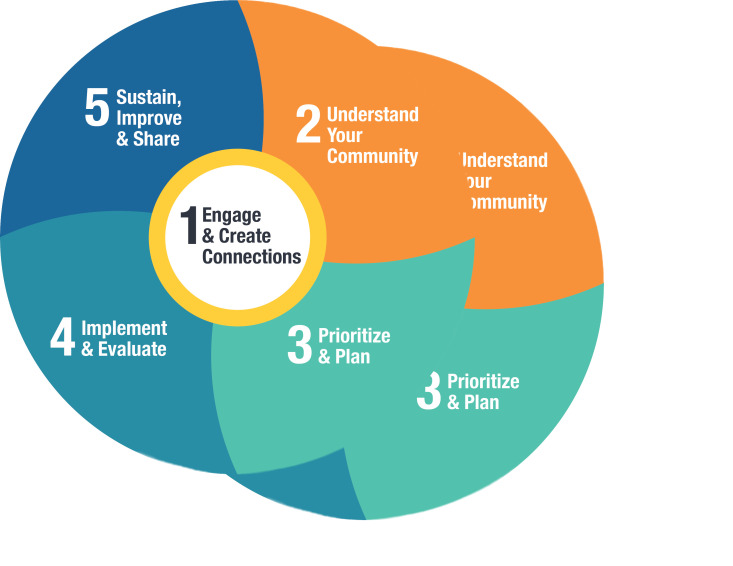
The AHCA iterative five-step process

**Source: **Primary Care Alberta.^18^

**Abbreviation: **AHCA, Alberta Healthy Communities Approach. 

Participating communities followed the AHCA process (Figure 1), which comprises five iterative steps: (1) engage and create connections; (2) understand your community; (3) prioritize and plan; (4)implement and evaluate; and (5) sustain, improve and share.^18^ Throughout the process, communities received implementation support in the form of mentoring by a CPSI health promotion facilitator (HPF); evidence-based tools and resources; and learning and sharing opportunities. Many resources are available online on the Alberta Healthy Communities Hub.^18^ Communities also received seed funding of CAD 20000 to develop and implement comprehensive efforts directed toward increasing community control over the determinants of health (i.e. healthy community initiatives), thereby creating supportive environments for health and cancer and chronic disease prevention. 

***Study design***

In this natural experiment study we utilized a multimethod design and process and a summative (i.e. outcome and impact) evaluation to determine the effectiveness of AHCA II. We used the RE-AIM evaluation framework^19^ to select indicators for the evaluation and to determine and synthesize project outcomes and impacts. In this paper we focus on effectiveness, but information on the other important evaluation findings (i.e. reach, adoption, implementation, maintenance) are available from the authors upon request. 

As this was an evaluation study, approval from an ethics committee was not sought. However, the ARECCI (A Project Ethics Community Consensus Initiative) Ethics Screening Tool^20^ was used to ensure minimal risks. The tool determined that the project posed “somewhat more than minimal risk” due to inexperienced project leads. A second opinion was sought (through virtual consultation), and the research team made sure that project leads were supported by experienced community-based evaluators to minimize risks to participants.

***Setting and participants***

The populations of interest were rural communities in Alberta with a concentrated population of 15000 or less in nonurban centres. Recruitment involved a call-out campaign from the CPSI Community Team to encourage eligible communities to submit a letter of intent to share their community’s story and motive for participating in the AHCA. After receipt of a letter of intent from a community, an HPF conducted an interview with the applicant(s) that focused on dimensions of AHCA readiness (e.g. existing supports, resources and collaborative practices). CPSI team members and partners then discussed each community’s application and the interviews, any prior experiences with the community, the communities’ existing capacity and how to make sure that the communities selected would be geographically diverse. 

AHCA II involved 19 rural communities that ranged in size from 522 to 14436 people (average size = 3767; median size = 2918) based on population counts from the 2021 Census of Population.^21^ In each community, multisectoral teams (MSTs) were formed and members collectively implemented the AHCA and participated in evaluation activities. The teams included members from diverse settings, including the community at large, community facilities and organizations, health care, schools and workplaces. MSTs included 4 to 30 individuals in the community, with a total of 258 individual MST members participating in the project.

***Data collection and analysis***

Both qualitative and quantitative data were collected with and from members of community MSTs. All MST members in AHCA II received a booklet that outlined the purpose of the project and provided detailed information on evaluation activities, the individual and community-level data collected, the risks and benefits of participating as a volunteer, privacy and confidentiality, and data management. All MST members were required to sign an informed consent form agreeing to the use of anonymized individual-level data for the purposes of knowledge translation (e.g. publications). After all the MST members had read the booklet and had had the opportunity to ask questions, a representative signed an informed consent form on behalf of the MSTs agreeing to the dissemination of community-level data. 

The sources of data used in this study were Community Capacity Assessment Tool (CCAT) results; Healthy Places Action Tool (HPAT) results; focus group transcripts; and results from a follow-up survey.

**Community Capacity Assessment Tool**

The CCAT is an evidence-informed assessment and planning tool that facilitates intentional in-depth consensus-building conversations concerning community capacity (i.e. the ability to address collective priorities).^22^ The MSTs discussed each of the questions across 11 domains (Table 1) and answered them using a five-point Likert scale from “haven’t started” to “we’re there” with corresponding scores of 1 to 5. A research associate [NF] then generated a report with the pre- and postimplementation scores for each CCAT domain for each MST.

**Table 1:** Community Capacity Assessment Tool domains that together indicate a community’s ability to address collective priorities

Domain	Description
1. Sense of community	Feelings of belonging and trust among community members.
2. Communication	Opportunities for people to share their ideas, knowledge and perspectives with others in order to bridge gaps, resolve conflicts and create effective ways of working together.
3. Partnerships, linkages and networks	The ability to form connections with diverse groups, organizations and individuals who share similar interests and goals.
4. Participation	Active and intentional engagement of community members, organizations and other partners throughout the initiative.
5. Resources	People, infrastructure, funding and time that can be leveraged to ensure the success and sustainability of community initiatives.
6. Skills and knowledge development	Opportunities to identify existing skills and gain new knowledge.
7. Asking why	Identifying the root causes of community concerns to design comprehensive solutions.
8. Learning from experience	Reflecting on and seeking feedback to understand what is working well and what can be improved upon to inform future action.
9. Shared vision	A detailed, realistic picture of the community that members strive to reach in the future.
10. Shared community leadership	Bringing together formal leaders and other people (or groups) for shared leadership.
11. Sustainability	Lasting benefits in a community through ongoing community action.

**Healthy Places Action Tool**

The HPAT is an evidence-informed planning and assessment tool that is used to identify and understand community settings and environments while focusing on established modifiable health behaviours for cancer and chronic disease (i.e. focus areas) (Table 2). It is used to identify community strengths and areas for improvement across various health domains as well as to prioritize areas for action to support community health and well-being. As with the CCAT, MSTs discussed and answered each of the corresponding questions using a five-point Likert scale to rank answers from “haven’t started” to “we’re there” from 1 to 5. A research associate [NF] then generated a report with the pre- and postimplementation scores for each HPAT setting, environment and focus area.

**Table 2:** Healthy Places Action Tool domains identifying community strengths and prioritizing community action

Setting	Environment	Focus area
Communities at large: The foundation for understanding the entire community.	Social: How people connect within the community, their cultures and their shared values.	Physical activity
Community facilities and organizations: The environments within community facilities, such as parks, libraries and community centres.	Physical: The community’s built and natural surroundings including the availability of walking paths, bike lanes and green spaces to encourage physical activity and access to nature.	Healthy eating
Health care: Hospitals, clinics and community health centres.	Economic: The affordability of health-related resources in the community.	Alcohol reduction
Workplaces: The environments in which people work, e.g. offices and factories.	Policy: The protocols and rules in place to support health and wellness.	Tobacco reduction
Schools: The environments that provide early, primary, secondary and postsecondary education.	n/a	UVR protection
n/a	n/a	Cancer screening

**Abbreviations: **n/a, not applicable; UVR, ultraviolet radiation. 

To evaluate effectiveness, an evaluation associate [JKKL] conducted inferential analyses on the pre- and postimplementation CCAT and HPAT data using paired sample *t*tests with effect size Cohen *d* scores to determine whether differences between before and after the completion of AHCA II were statistically significant.

**Focus groups**

Of the 19 MSTs, 18 participated in end-point impact focus groups; one MST had dissolved by the time the focus groups were conducted, and the members could not be contacted. The focus groups were conducted online via Microsoft Teams (Microsoft Corp., Redmond, WA, US) by one evaluation associate [JKKL] using a semistructured guide, between May 2023 and January 2024. One focus group was attended by only one MST member and was conducted as a semistructured interview. The largest focus group comprised 10 participants. A total of 86 MST members participated in 18 focus groups. 

Participants were asked about the AHCA’s impact in their community (example questions were as follows: “What was your vision of change for your community?”; “Did the AHCA help you achieve your vision?”; “What are the most significant changes you have seen in your community?”); community experience (e.g. “What tools and resources did you find most and least beneficial in achieving your vision?”); and sustainability plans (e.g. “How will you sustain and build on changes in your community?”).

Transcripts were verified for accuracy and anonymized. A research associate [CB] analysed the focus group transcripts using a “codebook” thematic analysis approach^23^ and NVivo qualitative analysis software version 12 (QSR International Pty Ltd., Melbourne, AU). A second researcher [CG] supervised the analysis process to ensure that interpretations reflected the dataset as a whole (i.e. peer debriefing).

**Survey of support partners**

After project completion, a survey was sent to support partners to ask about the impact and sustainability of the AHCA. Support partners were those in the MST who facilitated the AHCA process as employees or volunteers with community-level organizations (e.g. the recreation coordinator) and by working closely with a CPSI HPF. The online survey consisted of eight dichotomous (yes/no) and branching qualitative open-ended questions that focused on the sustainability of the MST (i.e. whether collaboration had been sustained and contributing factors); sustainability of healthy community initiatives (i.e. whether the initiatives had been sustained, whether new initiatives had been developed, and the contributing factors); and collective impact (i.e. whether the support partners had witnessed beneficial or harmful changes in the community since participating in the AHCA).

A link to the survey was emailed to the support partners by CPSI HPFs 6 months after communities completed the AHCA II. The survey responses were anonymous and collected via REDcap (Vanderbilt University, Nashville, TN, US). Upon receiving the survey link, support partners had 2 weeks to complete the survey; a follow-up email was sent if the survey was not completed within that time. The survey was sent to 18 support partners in 16 participating communities; a total of 15surveys were completed by support partners in 12 communities (response rate 83.3%). Conventional content analysis^24^ and NVivo 12 qualitative analysis software were used by a research associate [CB] to analyze the qualitative survey data. A second researcher [CG] supervised the analysis process and functioned as a peer debriefer.

***Data synthesis and reporting***

To determine the effectiveness of the AHCA, the four data sources (CCAT, HPAT, focus groups and follow-up survey) were reviewed and analyzed (i.e. triangulated) by a researcher [CG], a research associate [CB] and an evaluation associate [JKKL] involved in data collection and analysis. The team found that quantitative findings from the HPAT and CCAT results were contextualized by results from the qualitative focus groups and surveys, which provided additional perspectives concerning the effectiveness of the AHCA. The results were thus combined in a narrative synthesis to provide a comprehensive understanding of the summative outcomes and impacts of the AHCA. Identifiers accompanying illustrative quotations indicate that the participant is a support partner (SP) or community member (CM) who sat on an MST, along with the rural community they represented.

## Results

The evaluation results addressed three key outcomes and impacts of the AHCA in rural Alberta communities: improvements to supportive (built) environments for health; community wellness culture; and community capacity.

***Supportive (built) environments for health***

**Figure 2:**
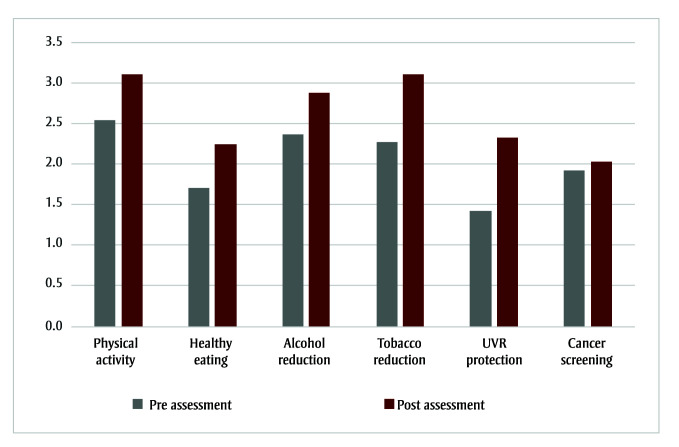
Mean assessment rating results for HPAT focus areas before and after implementation of AHCA II (n = 16)

**Abbreviations:** AHCA II, Alberta Healthy Communities Approach Phase II; HPAT, Healthy Places Action Tool; UVR, ultraviolet radiation. 

Of the 19 MSTs who completed a pre-HPAT assessment, 16 conducted a post-HPAT assessment. Nine MSTs completed a post-HPAT in full, and seven completed the post-HPAT for the focus areas identified as priorities for their communities. All six focus areas showed an overall improvement, from a mean preassessment rating (standard deviation [SD]) of 1.95 (0.43) to 2.70 (0.56) for the postassessment rating (*t*(15) = 4.45, *p* < 0.001, Cohen *d* = 1.11). The largest improvement was in “UVR protection,” from 1.43 (0.26) to 2.33 (0.83) on the rating scale (*t*(10)= 3.64, *p* < 0.01, Cohen *d* = 1.10). “Physical activity” grew from 2.53 (0.65) to 3.11 (0.64) (*t*(14)= 3.56, *p* < 0.05, Cohen *d* = 0.92), and “healthy eating” from 1.71 (0.44) to 2.25 (1.00) (*t*(15) = 2.38, *p* < 0.05, Cohen *d* = 0.60). “Tobacco reduction” changed from 2.27 (0.89) to 3.11 (1.30) (*t*(9) = 2.53, *p* < 0.05, Cohen *d* = 0.80). No statistically significant changes were found for “alcohol reduction” (2.37 [0.63]–2.89 [0.74]; *t*(10) = 1.56, nonsignificant [ns], Cohen *d* = 0.49) or “cancer screening” (1.93 [0.74]–2.03 [0.54], *t*(9) = 0.39, ns, Cohen *d* = 0.13) (see Figure 2).

To encourage physical activity, communities implemented changes to the built environment by building sledding areas, walking, cycling and cross-country skiing trails, disc golf courses and outdoor crokicurl and skating rinks; installing bike racks; and developing or enhancing parks and other outdoor play spaces including multisport, pickleball and basketball courts, skateboard and bike parks, and playgrounds. To support UVR protection, sunscreen stations and sun shelters were installed, shade trees were planted and signs about sun safety were posted. Communities promoted healthy eating by installing water filling stations, establishing community and youth gardens, making healthy food and water available in facilities and community spaces, and partnering with grocery stores to provide and promote healthy food options.

All but one of the communities (which focused only on physical activity) implemented initiatives that addressed more than one focus area; for example, one community installed a water filling station, enhanced an outdoor walking trail and built a gazebo for shade near the trail, while another established a community garden, installed bike racks, enhanced a playground and built sun shelters.

One participant explained that the community garden implemented as part of the AHCA had led to changes that support physical and social health and well-being: “it’s expanded to more than just feeding our community … the space is quite beautiful and with the sun shelters that are there, it’s become a meeting spot” [CM1, Wembley]. Another explained that a natural outdoor play space that included a garden, sunscreen stations and fruit and shade trees was encouraging healthy eating, physical activity, UVR protection and social connection:

The outdoor play space is being used much more than when it was just a green space. The school, daycare and [family and community support services] have added dedicated times to be outside in the space. This has brought about more gross motor, fine motor, risky and imaginative play to those that use the space. It has also produced fruit and vegetables that some of the families have never tasted before, so they were able to try new things. Plus, the increase in fresh air, sunshine, sunscreen usage and socializing as the families/individuals gather [SP3, Raymond]. 

The positive effects of supportive built environments were reported in other communities, particularly for the focus area of physical activity. One participant described their walking trail as well-used, which led “to an increase in physical activity and community connectedness” [SP6, Provost]. Another explained that their MST had established “[a] fitness trail […] to go along [with the] existing walking trail” [SP3, Bonnyville], which in turn promoted physical activity.

Alongside the physical changes to the built environment, communities created programming aimed at improving accessibility of health supports. For instance, communities implemented lending library programs that offered sports and physical activity equipment, health products, on-demand classes and cooking equipment that local residents could borrow. These programs also inspired community workshops and events such as cooking and preserving classes, seed exchanges and gardening events. One participant explained that the program “has been able to address several barriers that the community members face including not having access to equipment due to transportation issues, lack of funds and lack of exposure to opportunities” [SP11, New Sarepta]. Another stated that they had seen an “increase in outdoor recreation due to the [lending] library having items available to borrow instead of [having to purchase them]” [SP3, Raymond]. 

All but one of the support partners (n=14; 93%) said that the AHCA initiatives continued to be maintained for 6 months after the project ended. One mentioned that their town had maintained and expanded their outdoor skating rink “by adding seating, fire bowls, firewood and special event nights” [SP3, Raymond]. Another stated that “our community has continued on with building community connection by hosting different events/workshops promoting wellness” [SP13, Crossfield]. Other ways in which AHCA initiatives were built upon included purchasing new sporting equipment, expanding walking trails, building outdoor benches, planting trees and adding shade structures to existing parks. Communities also continued to implement programming within the wellness infrastructure developed through the AHCA, such as snowshoeing groups and drop-in sport programs.

***Community wellness culture***

Alongside changes to the built environment, AHCA II caused cultural shifts within communities that promoted both individual behaviour change and community health and well-being. When asked about the AHCA’s impact, participants said that the AHCA process had had the broader effect of creating a novel health and wellness culture within their respective communities. For example, one participant said that the AHCA process “created a stage for people [in the community] to champion mental health and well-being” [CM1, Valleyview]. Another stated that the AHCA had “created a wellness culture in the community. Community residents now seem to understand and participate in wellness-related activities” [SP2, Millet]. Yet another said that “many people still want to engage with the concept of healthy communities” [SP10, Grande Cache], indicating that the values-based approach had been accepted by and instilled in the culture of their rural community. One participant further noted that “there has been more open discussion about health and wellness” [SP13, Crossfield].

Alongside the collective sense of appreciation for and understanding of wellness, participants noted that community members were more engaged in healthy community initiatives. One participant said that the AHCA had “sparked interest in community wellness, [and] over time this has grown into community wellness events [such as mental health] initiatives, food security initiatives [and] overall community well-being” [SP2, Millet]. 

Modifications to the built environment had also led to new family activities and traditions and indicated changes to community ways of life. As one participant said, “skates are just flying off the shelves this year, and families are borrowing items all year long. One family lives along the walking path where the frisbee golf course is, so they borrow that set in order to play, especially when grandkids come over” [SP3, Raymond].

***Community capacity***

**Figure 3:**
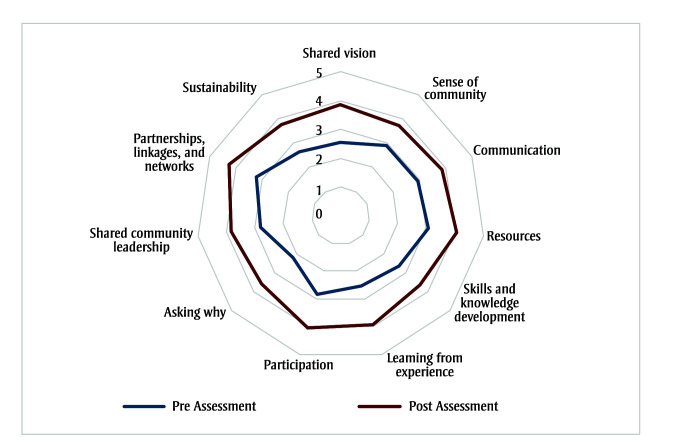
Mean CCAT results before and after participating in AHCA II (n = 17)

**Abbreviations: **AHCA II, Alberta Healthy Communities Approach Phase II; CCAT, Community Capacity Assessment Tool. 

Communities increased their capacity to address collective priorities and promote the health and well-being of their respective communities. After the implementation stage, 17 of the 18 MSTs who completed the CCAT preassessment also completed a postassessment; all 17 communities saw a significant increase in all 11 domains of community capacity on a scale of 1 to 5, from a mean (SD) preassessment rating of 2.76 (0.65) to 3.89 (0.56) for the postassessment rating (*t*(16) = 5.19, *p* < 0.001, Cohen *d* = 1.26) (Figure 3). The three top-rated domains were “partnerships, linkages and networks,” from 3.21 (1.08) to 4.27 (0.56) (*t*(16) = 3.42, *p* < 0.01, Cohen *d* = 0.83); “resources,” from 3.08 (1.07) to 4.08 (0.80) (*t*(16) = 3.49, *p* < 0.01, Cohen *d* = 0.85); and “participation,” from 2.82 (1.03) to 4.05 (0.57) (*t*(16) = 3.99, *p*= 0.001, Cohen *d* = 0.97). Improvements were also observed in “asking why,” from 2.19 (0.81) to 3.61 (0.89) (*t*(16) = 4.57, *p* < 0.001, Cohen *d* = 1.11), with an emphasis on critical thinking and questioning in community capacity-building. “Learning from experience” showed a significant improvement, from 2.55 (0.82) to 3.95 (0.68) (*t*(16) = 4.86, *p* < 0.001, Cohen *d*= 1.18), as did “shared vision,” from 2.56 (1.12) to 3.87 (0.63) (*t*(16) = 3.42, *p* < 0.01, Cohen *d*= 1.25).

In line with the CCAT results, participants said that the AHCA had established and/or strengthened multisectoral collaborations and partnerships, improved community participation and increased resources. One participant explained that the AHCA had facilitated access to resources as well as “ways to meet other people and get partnered up with things” [CM1, Hanna], while another said, “I think the knowing of individuals and organizations and pulling them all together into the same room has been hugely beneficial” [CM1, Langdon]. Another participant explained that “so many different groups came together, people representing different areas came together and are working together to push together to make change, rather than everybody working on separate projects” [CM2, Brooks]. Participants also noted that the AHCA had helped to strengthen relationships across communities in rural Alberta. As one participant noted, “the connections inside [our community], which I’ve seen from throughout this committee, are, I would say, the most valuable, but also as a secondary, getting connections with other communities as well” [CM3, Raymond].

Participants also recognized the positive impact of the AHCA on encouraging community participation. The process of community engagement resulted in meaningful participation of individuals and groups within the community, which helped to establish and maintain changes to the built environment. For instance, participants noted that their communities had more residents volunteering their time. One participant stated that “over the course of the last 4 years, I think [community members’] participation on the community team has skyrocketed” [CM1, Jasper]. 

As an example of community participation, another participant mentioned that “families help plant the gardens in the spring and harvest them in the fall…. Community members are donating items to the lending library,” and that their community specifically engages youth in an outdoor play space “by creating signage, decor and stepping stones, and helping with cleanup and maintenance” (SP3, Raymond). By actively engaging community members, organizations and other partners, communities were enabled to lead initiatives. One participant reflected that

… there’s almost a courage in the community … community members now are taking their ownership and their own leadership, and they’ve built a strength to be able to do it on their own [CM7, Millet]. 

Communities also reflected that the AHCA had increased their ability to identify and leverage existing resources, including people, infrastructure and funding. One participant shared that their community had “built on what was already so great about [our town] and made it even better,” [CM6, Millet] while another explained that “there’s a lot of people that are willing to share their knowledge and expertise and talents, which I think really drives a lot of the projects” [CM3, Langdon]. Similarly, a participant described the importance of leveraging resources to increase community capacity:

At the end of the day nothing beats real live bodies inside a room, with ideas, like building relationships and really like centring those relationships… I think a lot of times we externalize things and we go looking outside for solutions, which is also important. But the wisdom and the talent and the ideas and the people who understand the context … are right here inside of these organizations already. They’re right here in the towns that we live in, in the businesses that we run, and we can actually lean on each other, support each other, and be there for one another as a community [CM1, Edson]. 

To support and expand their initiatives, communities leveraged the data and resources generated from the AHCA to secure additional funding and in-kind contributions. When reflecting on the unexpected impacts of the AHCA, one participant said:

I think organizing ourselves a bit more and, like, selling the benefit of this approach to social change might have elevated the profile of what we’re doing with our elected officials and thus created more opportunities for funding and local government buy-in [CM1, Jasper]. 

Communities also recognized that their increased capacity to leverage resources supported the sustainability of their healthy community initiatives. As one community member said:

I don’t worry [about when] the funding and stuff is done because I feel like there’s so much already embedded in the community. That, and I mean it needs to be community led. That’s how things work is when the community leads it [CM7, Millet]. 

## Discussion

The aim of this study was to evaluate and describe community-level outcomes and impacts of the AHCA II. The evaluation with the 19 MSTs who implemented the AHCA in rural communities found that the process resulted in changes to the built environment and positive short-term outcomes related to primary cancer and chronic disease prevention.

Most research on the built environment and health outcomes and behaviours in Canada is focused on urban areas, and health-related initiatives often fail to account for the unique demographic characteristics and social, cultural and economic conditions of rural communities.^3^,^5^,^12^ Recognizing the diversity within and between rural places, the AHCA is a flexible process that can be tailored to the specific needs, strengths and determinants of health in different rural communities. This study provides an example of an approach that resulted in improved built environments and in shifts in cultural values and community capacity to promote and sustain healthy behaviours and community development.

Using the AHCA, MSTs collaborated to create and/or enhance environments that promote and support healthy behaviours in their respective rural communities. The evaluation was based on self-reported data and was not designed to objectively capture changes in individual-level health outcomes (e.g. waist circumference) or behaviours (e.g. fruit and vegetable intake). Nevertheless, community members reported that improvements to the built environment increased healthy behaviours. Programming further supported these changes in behaviours by helping to address barriers to accessing and/or affording health-promoting resources.

Although the connections are complex, researchers have demonstrated the reciprocal links between the built environment and individual health behaviours, which in turn, influence individual and population health outcomes.^4^,^25^,^26^ Our findings support the evidence that changes to the built environment in rural areas can positively influence health behaviours and outcomes by providing opportunities, resources and supports for community members.^26^,^27^ For instance, a recent study found that bike lanes, pedestrian safety features and community beautification were inversely associated with obesity in a cohort of individuals from urban and rural communities in 21 different countries.^28^

In this study, rural MSTs designed and implemented a number of initiatives related to healthy eating, physical activity and UVR exposure, based on community-identified priorities. A scoping review that did not consider community type found that transportation, housing and spatial accessibility influence key risk factors for cancer such as air quality, diet and physical activity.^29^ These factors may disproportionately affect rural areas, which generally have more hazardous environmental conditions, a lack of facilities and infrastructure, and poorer health service availability.^7^,^30^ As such, achieving long-term impacts on cancer and other chronic diseases may require thinking beyond the physical elements of rural communities and assessing and addressing the wider set of resources, conditions and built environment features that impact residents’ health behaviours and outcomes. For instance, ensuring that community members can access safe and affordable transportation, housing and health services through community design and land use planning may serve to improve the built environment in ways that optimize health outcomes.^31^

The findings of this evaluation also indicate that the AHCA implementation resulted in outcomes and impacts that indirectly relate to the built environment. The MSTs observed important cultural changes in their communities, including those related to perceptions of health, willingness to support community activities and events, and family traditions that incorporate health-promoting behaviours. The combined effects of tangible enhancements to the built environment, supportive programming and shifting attitudes on health and well-being enabled community members to engage more frequently in physical activity, adopt healthier eating habits and prioritize overall wellness. This suggests that the participating communities embraced the value system associated with the AHCA, which is critical for implementing specific health-promoting initiatives and reducing health inequity.^32^,^33^ Given that cultural elements—including values, beliefs, norms and practices concerning health and well-being—are core to rural ways of life, changes to the sociocultural environment within rural communities can promote or inhibit health equity at a local level.^5^,^34^,^35^ When reinforced by the built environment, cultural changes in communities thus have the potential to further influence actual health behaviours and reduce risk of cancer and other chronic diseases in the longer term.

Although these findings are encouraging, making widespread changes to the built environment is difficult and observing the related health outcomes takes considerable time and effort. Given the complexity of social and physical environments, it is also challenging to link cancer and chronic disease prevalence to specific aspects of the built environment. Nevertheless, there is a need for rigorous evaluations of community initiatives that modify the built environment, in different contexts, to determine whether and to what extent they result in health outcomes as well as to determine why and for whom they are effective (or ineffective). While longitudinal study designs are ideal, they are not always possible in community settings because of time, capacity and resource constraints. However, natural or quasi-experiments like the one in this study are appropriate for evaluating the efficacy of built environment changes on health outcomes.^36^ To support the design of strong studies, conceptual frameworks that consider the built environment and its impact on health^37^-^39^ may help identify features of the built environment that mediate modifiable health behaviours for cancer and chronic disease, guide the evaluation of health outcomes based on comprehensive indicators and determine the mechanisms behind any effects.

Finally, this evaluation demonstrated that the AHCA improved community capacity to achieve supportive environments for health through multisectoral collaborations, community participation and leveraging resources. Rural communities generally have poorer access to funding, infrastructure and human capital, which can influence their capacity to initiate and sustain health-promoting initiatives.^5^,^12^,^40^,^41^ Previous research and strategic recommendations for rural health equity focused on leveraging formal and informal skills of community members, using local resources and existing infrastructure and building multidisciplinary and multisectoral collaborations to make environmental changes and create opportunities for rural residents to engage in healthy behaviours.^5^,^12^,^41^,^42^ Indeed, our study found that the AHCA helped bring community members, organizations and other partners together to enhance the built environment through creative solutions that leveraged collective strengths and resources.

Innovative and creative ways of working that actively engage and connect diverse community members and other partners (e.g. mayors and municipal leaders, community support services staff, health care personnel, youth leaders, school officials, librarians, business owners, religious leaders) are required in order to foster long-term changes to the built environments that shape rural areas and influence residents’ health behaviours. Although one of the MSTs that participated in AHCA II had dissolved by project end, the remaining 18 had sustained their collaboration and their healthy community initiatives. This suggests that the AHCA is a sustainable platform for engaging and enabling individuals from different sectors, groups and organizations to facilitate action at the local level and promote health and ultimately improve cancer and chronic disease outcomes. Nevertheless, it is crucial for all MSTs to consider how to sustain collaborations and partnerships from the earliest stages and throughout the AHCA process. The availability of funding, staff and volunteers must also be considered and accounted for to support maintenance of healthy community initiatives aimed at improving the built environment.

***Strengths and limitations***

This project was conducted in collaboration with communities to build capacity to develop, implement and evaluate health promotion initiatives. We utilized a rigorous evaluation design as well as multiple measures and data sources to determine effectiveness. However, it is important to consider the impact of the COVID-19 pandemic on the AHCA II evaluation given that a state of public health emergency was declared in Alberta about a year into the project. Communities exhibited considerable resilience in navigating pandemic-related challenges to participate in evaluations,^43^ but were dealing with many social and economic issues when completing the HPAT and CCAT assessments. These circumstances resulted in significant MST turnover, and in one case, forced them to withdraw from the AHCA II. Communities also had to transition to online collaboration in the middle of completing assessments. As a result, not all communities completed pre- and post-HPAT and CCAT assessments in full (or at all). Overall, these unique circumstances may have inadvertently skewed findings so that they did not reflect all community experiences or were not representative of the contextual conditions influencing AHCA outcomes.

Despite these potential limitations, the evaluation reflects the strengths, priorities and characteristics of diverse rural communities in Alberta. The transferability of the findings to other communities will depend on whether other communities (and researchers) determine if and how they apply to the new settings. However, the observational study design we used limits the potential to detect causal influences and mechanisms as well as health outcomes. Additional longitudinal studies are necessary to determine which features of the built environment cause changes in health, the mechanisms and mediators of change, and whether there are differential effects on diverse populations over time. As others have suggested, future studies could use causal inference methods to allow for associations between community environments and cancer and chronic disease outcomes.^44^

It is also important to note that an equity lens was not included in the evaluation design, and we do not know whether and to what extent the changes to the built environment were acceptable to, equally available to and utilized by all the individuals within the participating communities. It is therefore possible that there were negative consequences of the built environment changes but that these were not reported. Our team is currently adapting the AHCA to include a stronger focus on promoting and improving equity in urban contexts, and this adaptation will also be evaluating equity outcomes.

## Conclusion

This evaluation has demonstrated that the AHCA results in positive short-term outcomes within and across rural communities in Alberta. In addition to improving supportive environments for health, the AHCA has facilitated cultural changes and improved community capacity. Each of these components is required to support long-term behaviour change to promote health and prevent and reduce incidence of cancer and chronic disease. While the positive changes reported in rural communities are encouraging, it takes time to determine whether changes to the built environment and individual health behaviours are maintained. Seeing the individual, community and population-level health effects of these changes also requires considerable capacity to systematically collect and share evidence over time. Long-term follow-up is required to determine whether changes initiated by healthy community approaches like the AHCA are sustained within rural communities and result in positive behaviour changes that shape healthy futures characterized by lower incidence of cancer and chronic disease.

## Acknowledgements

The authors thank the following communities for their participation in this study: Barrhead, Bonnyville, Brooks, Crossfield, Edson, Grande Cache, Hanna, Jasper, Langdon, Millet, New Sarepta, Provost, Raymond, Sexsmith, Smoky Lake, Stettler, Thorsby, Valleyview and Wembley. We also thank our dedicated and passionate team of seven health promotion facilitators, whose community health–promotion expertise was essential to the Alberta Healthy Communities Approach process: Beverly Milroy, Cheryl Stetsko-Mayne, Magan Braun, Molly Hanson-Nagel, Shana Young, Teree Hokanson and Yvonne Rempel. Zone and provincial teams from Alberta Health Services provided valuable expertise, insight and partnership. Strategic direction and applied research support was provided by the Cancer Prevention and Screening Innovation team. 

## Funding

Funding was provided, in whole or in part, by Alberta Health. Provision of funding by Alberta Health does not signify that the findings represent the policies or views of Alberta Health.

## Conflicts of interest

None.

## Authors’ contributions and statement

CG: Conceptualization, formal analysis, methodology, project administration, supervision, writing—original draft, writing—review and editing.

LKAS: Conceptualization, project administration, writing—review and editing.

CB: Formal analysis, writing—review and editing.

NF: Data collection, writing—review and editing.

JKKL: Data collection, formal analysis, writing—review and editing.

SP: Conceptualization, project administration, writing—review and editing.

The content and views expressed in this article are those of the authors and do not necessarily reflect those of Primary Care Alberta or the Government of Canada.

## References

[B01] Catalogue no Health of Canadians [Internet]. Statistics Canada.

[B02] Koohsari MJ, Nakaya T, McCormack GR, Oka K (2021). Built environment design and cancer prevention through the lens of inequality. Cities.

[B03] Tam T The Chief Public Health Officer’s report on the state of public health in Canada 2017: designing healthy living. Public Health Agency of Canada.

[B04] Gelormino E, Melis G, Marietta C, Costa G (2015). From built environment to health inequalities: an explanatory framework based on evidence. Prev Med Rep.

[B05] Leimbigler B, Li EP, Rush KL, Seaton CL (2022). Social, political, commercial, and corporate determinants of rural health equity in Canada: an integrated framework. Can J Public Health.

[B06] Matz CJ, Stieb DM, Brion O (2015). Urban-rural differences in daily time-activity patterns, occupational activity and housing characteristics. Environ Health.

[B07] Lavergne MR, Kephart G (2012). Examining variations in health within rural Canada. Rural Remote Health.

[B08] Williams AM, Kulig JC, Kulig JC, Williams AM (2011). Health and place in rural Canada. UBC Press.

[B09] Canadian Cancer Statistics: a 2022 special report on cancer prevalence [Internet]. Canadian Cancer Society.

[B10] Lung cancer and equity: a focus on income and geography. Canadian Partnership Against Cancer.

[B11] DesMeules M, Pong RW, Guernsey JR, Wang F, Luo W, Dressler MP, Kulig JC, Williams AM (2011). Rural health status and determinants in Canada. UBC Press.

[B12] Hansen AY, Meyer MR, Lenardson JD, Hartley D (2015). Built environments and active living in rural and remote areas: a review of the literature. Curr Obes Rep.

[B13] Seguin R, Connor L, Nelson M, LaCroix A, Eldridge G (2014). Understanding barriers and facilitators to healthy eating and active living in rural communities. J Nutr Metab.

[B14] Hancock T (2014). The little idea that could: a global perspective on healthy cities and communities. Natl Civ Rev.

[B15] Hancock T, Norris T, Lacombe R, Perkins F, Leeuw E, Simos J (2017). Healthy cities and communities: the North American experience. Springer.

[B16] Williams-Roberts H, Jeffery B, Johnson S, Muhajarine N (2016). The effectiveness of healthy community approaches on positive health outcomes in Canada and the United States. The effectiveness of healthy community approaches on positive health outcomes in Canada and the United States. Soc Sci (Basel).

[B17] Chaisson K, Gougeon L, Patterson S, Scott LK (2022). Multisectoral partnerships to tackle complex health issues at the community level: lessons from a Healthy Communities Approach in rural Alberta, Canada. Can J Public Heal.

[B18] Alberta Healthy Communities Approach [Internet]. Primary Care Alberta.

[B19] Glasgow RE, Vogt TM, Boles SM (1999). Evaluating the public health impact of health promotion interventions: the RE-AIM framework. Am J Public Health.

[B20] ARECCI Ethics Screening Tool [software]. Alberta Innovates.

[B21] Census profile, 2021 Census of population. Statistics Canada.

[B22] Chaisson K, Braun M, Hokanson T, Rempel Y, Young S, Gougeon L, et al (2025). Understanding and building community capacity through conversation: a conversation forward Community Capacity Assessment Tool (CCAT) to catalyze action. J Rural Community Dev.

[B23] Braun V, Clarke V (2021). Thematic analysis: a practical guide. Sage Publications.

[B24] Hsieh HF, Shannon SE (2005). Three approaches to qualitative content analysis. Qual Health Res.

[B25] Turnbull R (2021). Healthy, happy places—a more integrated approach to creating health and well-being through the built environment. Br Med Bull.

[B26] Wilkie S, Townshend T, Thompson E, Ling J (2019). Restructuring the built environment to change adult health behaviors: a scoping review integrated with behavior change frameworks. Cities Health.

[B27] ller C, Paulsen L, Bucksch J, Wallmann-Sperlich B (2024). Built and natural environment correlates of physical activity of adults living in rural areas: a systematic review. Int J Behav Nutr Phys Act.

[B28] Corsi DJ, Marschner S, Lear S, Hystad P, Rosengren A, Ismail R (2024). Assessing the built environment through photographs and its association with obesity in 21 countries: the PURE Study. Lancet Glob Health.

[B29] Wray AJ, Minaker LM (2019). Is cancer prevention influenced by the built environment. Cancer.

[B30] Chrisman M, Nothwehr F, Yang G, Oleson J (2015). Environmental influences on physical activity in rural Midwestern adults: a qualitative approach. Health Promot Pract.

[B31] Kim MO, Montemurro G, Nieuwendyk L, Nykiforuk CI (2023). Supporting healthy community decision-making in municipalities: a synthesis of evidence-informed resources from across Canada. Wellbeing Space Soc.

[B32] Leeuw E (2012). Do healthy cities work. J Urban Health.

[B33] Leeuw E, Simos J, Leeuw E, Simons J (2017). Healthy cities move to maturity. Springer.

[B34] Rosal MC, Wang ML, Silfee VJ, Hilliard ME, Riekert KA, Ockene JK, Pbert L, th ed (2018). Culture, behavior, and health. The handbook of health behavior change.

[B35] Seguin-Fowler RA, Hanson KL, Villarreal D, Rethorst CD, Ayine P, Folta SC, et al (2022). Evaluation of a civic engagement approach to catalyze built environment change and promote healthy eating and physical activity among rural residents: a cluster (community) randomized controlled trial. BMC Public Health.

[B36] Mayne SL, Auchincloss AH, Michael YL (2015). Impact of policy and built environment changes on obesity-related outcomes: a systematic review of naturally occurring experiments. Obes Rev.

[B37] Solar O, Irwin A A conceptual framework for action on the social determinants of health. World Health Organization.

[B38] Frank LD, Iroz-Elardo N, MacLeod KE, Hong A (2019). Pathways from built environment to health: a conceptual framework linking behavior and exposure-based impacts. J Transp Health.

[B39] Fan Y, Song Y (2009). Is sprawl associated with a widening urban-suburban mortality gap. J Urban Health.

[B40] Sibley LM, Weiner JP (2011). An evaluation of access to health care services along the rural-urban continuum in Canada. BMC Health Serv Res.

[B41] Barnidge EK, Radvanyi C, Duggan K, Motton F, Wiggs I, Baker EA, et al (2013). Understanding and addressing barriers to implementation of environmental and policy interventions to support physical activity and healthy eating in rural communities. J Rural Health.

[B42] Edwards MB, Theriault DS, Shores KA, Melton KM (2014). Promoting youth physical activity in rural southern communities: practitioner perceptions of environmental opportunities and barriers. J Rural Health.

[B43] Gillies C, Frenette N, Patterson S, Scott LK (2024). Healthy community initiatives in rural Alberta, Canada, during COVID-19. Healthy community initiatives in rural Alberta, Canada, during COVID-19. J Rural Community Dev.

[B44] Gomez SL, Shariff-Marco S, Derouen M, Keegan TH, Yen IH, Mujahid M, et al (2015). The impact of neighborhood social and built environment factors across the cancer continuum: current research, methodological considerations, and future directions. Cancer.

